# Effects of vigor at work and weekly physical activity on job stress and mental health

**DOI:** 10.1038/s41598-022-19966-z

**Published:** 2022-09-26

**Authors:** Daniel Cortés-Denia, Sandrine Isoard-Gautheur, Esther Lopez-Zafra, Manuel Pulido-Martos

**Affiliations:** 1 Department of Psychology, School of Humanities and Sciences of Education, University of Jaén, Jaén, Spain; 2 Laboratoire Sport et Environnement Social, Université Grenoble Alpes, Grenoble, France

**Keywords:** Psychology, Health care

## Abstract

This study explores the role of personal resources, specifically vigor at work (a positive affect generated by the ongoing interactions in the workplace) and weekly physical activity (PA), in the stress-mental health relationship, given the positive relationships found between PA and levels of vigor experienced on health. Thus, we hypothesized that vigor at work would mediate the relationship between job stress and workers' mental health, whereas weekly PA would moderate the relationship between stress and vigor at work. Five hundred and twenty-seven workers completed self-report scales for stress, weekly PA, vigor at work, and mental health. The results showed that vigor at work was related to better mental health, whereas stress was related to high psychological problems and low vigor at work. The interaction between stress and weekly PA on vigor was significant, indicating a counterproductive effect of weekly PA. Specifically, the negative relationship between stress and vigor at work was greater when doing weekly PA. In this vein, high levels of weekly PA would not have a favorable impact when workers experience high levels of stress, consuming part of vigor at work and reducing the positive effect of vigor at work on mental health by coping with stress.

## Introduction

The European Agency for Safety and Health at Work^1^ has found that the 49% of Spanish workers indicate that stressful situations are common in their workplaces. This value is similar to the European average (51% of workers in Europe), where 27 member states of the European Union and 4 members of the European Free Trade Association participated. Moreover, 17% of the workers report having experienced stress daily at work^2^. In this sense, the World Health Organization (WHO)^3^ indicates that work-related stress is a major problem in the area of occupational health and safety, becoming the most important perceived occupational risk (53% of respondents)^4^. In addition, job stress relates to a higher risk of poor mental health^5,6^, as well as higher levels of anxiety and depression^7^. Therefore, workers´ mental health should be given greater importance^8^. However, potential mediators in this relationship may attenuate the effect of stress on health. For example, we posit that an affective personal resource, such as vigor at work^9,10^, could mediate the relationship between stress and mental health of the workers, analyzing whether physical activity (PA) may play a role, as a moderating variable, between stress and vigor at work. Hence, the main aim of this study was to examine the mediating role of vigor, and the moderating role of PA on the stress-mental health relationship at work.

### Stress-mental health relationship

The WHO^11^ has defined work-related stress as “the response people may have when presented with work demands and pressures that are not matched to their knowledge and abilities and which challenge their ability to cope” (para. 1). The job demands control model (JDC)^12,13^ explains how different job characteristics can influence the psychological well-being of employees. This model postulated that the interaction between psychological job demands and control at work would foster stress. In this model, psychological job demands are represented by those psychological demands that the work generates on workers (i.e. volume of work, level of attention and concentration, deadlines to deliver the work, …), and control at work is represented by autonomy (ability to make decisions) and skill development. Specifically, Chela-Alvarez et al.^14^ found that work overload, time pressure, low control, and lack of sufficient resources have been the most important factors to explain stress levels in the workplace. Hence, high psychological demands levels with low levels of control at work will lead to higher levels of psychological tension and stress, which can, in turn, generate a greater risk of mental illness, when it is prolonged in time.

According to the WHO^15^, mental health has been defined as "a state of well-being in which the individual realizes his or her abilities, can cope with the normal stresses of life, can work productively and fruitfully, and can make a contribution to his or her community" (p.1). Thus, the strains at work, due to the imbalance of psychological demands and job control at work, prevent good functioning in the worker, making an association between stressful work conditions with poor mental health^16^. In this sense, a systematic review and meta-analysis performed by Theorell et al.^17^ found that high psychological demands and low control at work had a significant impact on the development of depressive symptoms, whereas having a high control at work is a protector of depressive symptoms. Moreover, job stress can not only precipitate depression but also leads to high levels of anxiety^18^, as well as a worse sleep quality^19^. Thus, the use of multiple indicators is necessary to provide a more complete picture of mental health needs than a single indicator^20^. For that reason, research should consider a broader perspective of the mental health concept, taking into account further indicators to obtain a more complete picture of this concept. Hence, having a better construction of mental health will allow knowing in greater depth the effect that stress, through the imbalance of psychological demands and job control, has on it. Therefore, it would be relevant to include, in addition to depression, anxiety, and insomnia, the presence of social dysfunction and somatic symptoms that may explain, more accurately, the global mental health concept.

### Role of vigor at work in the stress-mental health relationship

Given the influence of stress on workers´ mental health, it therefore would be relevant to look at the protective variables that could attenuate this relationship. Consequently, this could enable organizations to promote these protective variables to improve the mental health and well-being of workers. Hence, it is possible to analyze the influence of personal resources, through the conservation of resources theory (COR)^21^. Personal resources (such as organization-based self-esteem, self-efficacy…) are the psychological aspects, associated with resilient processes, and refer to the ability to control and act on the environment in an adaptive way^22^. According to the COR theory, people are motivated to obtain, protect and promote different personal, social and material resources, which are highly valued and essential to avoid tensions^21^, helping people to manage stressors in the workplace. For example, according to this theory, stress would be the result of the inability to obtain resources, resource loss or the threat of resource loss. Therefore, personal resources may have an important role in the stress-tension framework, mediating the relationship between stress and health. Thereby, vigor at work^9^, which stems from COR theory, could be a fundamentally positive affect to protect and promote the health of workers. In this venue, vigor at work, as a personal resource, could promote organizational commitment, learning and the building of skills^9^ to face different situations or difficulties at work. Vigor at work^9,10^ is a positive affect generated by the various ongoing interactions in the workplace. It is composed of three dimensions: Physical Strength, which represents the physical capacities of the individual; Cognitive Liveliness regarding mental agility and the flow of thought processes; and Emotional Energy referring to the ability to show and express compassion and empathy to other people^9^.

Regarding the stress-vigor relationship, Shirom^10^ assumed that the decrease of energy in a worker may be the result of chronic and repeated exposure to job stress, reducing the worker's cognitive ability to process information. However, few studies have analyzed this relationship. For example, Malik et al.^23^ found that job stress was negatively related to vigor, concerning energy, resilience, persistence, and effort, but not exclusively measured at the cognitive, behavioral, and emotional level^9,10^. On the other hand, based on the relationship between vigor-mental health, some studies have found negative relationships between vigor, and insomnia, and symptoms of depression^24,25^. Therefore, stress experienced by workers will lead to a decrease in vigor, which has been shown to protect mental health in the workplace. However, no studies have previously analyzed vigor at work, as a mediator, in the relationship between of the relationship between stress and mental health.

### Role of PA in the stress-vigor relationship

Following the COR theory, vigor at work may be within a “positive profit spiral” of resources, where different positive resources can reinforce each other forming over time in profit spirals^26^. In this line, vigor at work may be influenced by other personal resources of workers. For instance, experiencing vigor has been one of the reasons for doing PA^27^. Several studies have found a positive relationship between PA and levels of vigor experienced^28,29^ and other positive affects (i.e. positive activated affect)^30,31^. In these studies, PA has been raised as an antecedent. Nevertheless, the mechanisms or the role of personal resources are not limited to acting as antecedents. In this vein, Schaufeli and Taris^32^ indicated that personal resources could have different roles (i.e. antecedents, mediators, moderators, etc.), not being mutually exclusive, and they all have empirical support, thus, no one is better than another. Scotto di Luzio et al.^33^ suggested that weekly PA, as a moderator, can increase workers' earnings and resources recovery, thereby increasing the experience of vigor in the workplace. In this vein, other studies have conceptualized PA as a moderating personal resource in the relationship between stress and exhaustion^34–36^ and further results related to wellbeing^37^. Nevertheless, although these studies found that PA positively moderates the relationship between stress and exhaustion and, thus, a positive quality can be attributed to PA, this moderating role may not always result in a beneficial (buffering) effect. In fact, Fodor et al.^38^ only found that weekly vigorous PA moderates the relationship between demands and exhaustion. Consequently, we wonder if including less vigorous forms of PA would make any difference.

**Figure 1:**
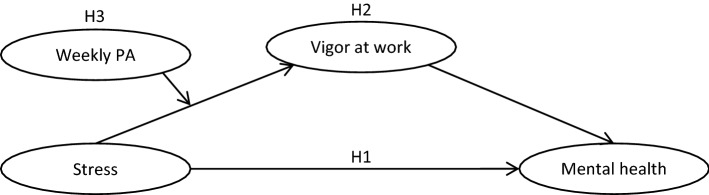
Hypothesized moderated mediation model.

Bearing all the above in mind, this study aims to enhance insight into the relationship between job stress and workers' mental health, testing the fit to the data of a moderated mediation model, where vigor at work mediates the relationship between job stress and workers' mental health, whereas PA moderates the relationship between stress and vigor at work. Specifically, we propose (see Fig. 1):

#### *Hypothesis 1.*

Stress will be related to lower mental health, as evaluated through measures of Somatic Symptoms, Anxiety/Insomnia, Social Dysfunction, and Depression.

#### *Hypothesis 2.*

Vigor at work will mediate the relationship between stress and mental health. Specifically, stress will be negatively related to vigor at work; and vigor at work will be positively related to better mental health.

#### *Hypothesis 3.*

Employee PA levels will moderate the strength or intensity of the indirect relationship between stress-mental health through experienced levels of vigor at work, such that the relationship is more (or less) intense when PA levels of employees are higher (or lower).

## Methods

### Participants and Procedure

Initially, five hundred and ninety-four volunteer employees completed a cross-sectional online survey with self-reported measures. However, sixty-seven employees were eliminated due to outliers responses or to detected inattention to a control item included^39^. Accordingly, five hundred and twenty-seven employees (53.3% were female), with a mean age of 38.2 years (SD = 11.5; range 19–63 years) and an average tenure in the organization of 9.9 years (SD = 10; range 0.5–41 years), composed the final sample. Regarding the type of organization, 62% of the workers were from private organizations, 31.9% from public organizations, and 6.1% from mixed organizations. The main working sectors were education and training (15.9%), economics and business administration (15.6%), informatics and telecommunications (15%), healthcare (11.2%), industry, mechanics, electricity and electronics (8.2%), social sciences (6.6%), communication, advertising and public relations (6.3%), and hotels, catering and tourism (4.6%); the rest of the sectors were less than 4%. The workers had university studies (44.6%), high-school studies (23.1%), postgraduate studies (15.7%), primary studies (12%), secondary studies (4.4%), and without studies (0.2%). The participants were obtained through a convenience sample and were recruited, between October 2020 and January 2021, by undergraduates from psychology, social education, and social work in the first author's university, who were instructed about the procedure, the inclusion criteria to participate (employees with tenure in the organization for at least six months) and the distribution of the survey.

### Ethical approval and consent to participate

The protocol was approved by the Ethics Committee of the University of Jaén, Spain (Ref. NOV.19/1.PROY). This research was performed following relevant guidelines and regulations. After an explanation of the aims and protocol of this study to each participant, written informed consent was obtained.

### Measures

*Stress* was measured with the Job Content Questionnaire (JCQ^13^; Spanish version by Escribà-Agüir et al.^40^) through the imbalance between psychological demands and control at work dimensions. These two subscales are composed of 13 items distributed for Psychological Demands (6 items; i.e. “My job requires working very fast”) and Control at Work (7 items; i.e. “My job requires a high level of skill”), with 4-point Likert response format from 1 (*strongly disagree*) to 4 (*strongly agree*). Cronbach’s alpha for the two subscales, in this study, were 0.71 and 0.81 respectively. The recommendations of Karasek and Theorell^13^ were followed to calculate the stress ratio, but for the items in the Spanish version. Values greater than 1 indicate “stress” whereas values under 1 indicate “not stress”.

*Physical activity* was measured with the International Physical Activity Questionnaire-Short Form (IPAQ-SF; Craig et al.^41^). This instrument is composed of 7 items with a numeric response format (i.e. “During the last 7 days, how many days did you do vigorous physical activities like heavy lifting, digging, aerobics, or fast bicycling?”). Thus, it evaluates the days and the number of minutes spent for each category of PA (walking, moderate and vigorous), during the last 7 days. This evaluation allows calculating the total PA for the week, generating a total weekly PA score based on the energy requirements of each activity, which is expressed in metabolic expenditure (METs) in minutes per week. These METs-minutes per week can be calculated by multiplying the days and the minutes, of each category, by 3.3 for walking; 4 for moderate activity; and 8 for vigorous activity. This allows obtaining a total score by adding the three categories of activity, for each worker, where high values indicate high total weekly PA and, therefore, a high metabolic and energetic expenditure per week.

*Vigor at work* was measured with the Shirom-Melamed Vigor Measure (SMVM^9^; Spanish version by Pulido-Martos et al.^42^). This instrument is composed of 12 items and comprises three subscales: Physical Strength (5 items; i.e. “I feel energetic”), Cognitive Liveliness (3 items; i.e. “I feel I am able to contribute new ideas”), and Emotional Energy (4 items; i.e. “I feel able to be sensitive to the needs of coworkers and customers”), with a 7-point Likert response format from 1 (*almost never*) to 7 (*almost always*). Cronbach’s alpha for the three subscales, in this study, were 0.94, 0.85, and 0.85 respectively.

*Mental health* was measured with the General Health Questionnaire (GHQ-28^43^; Spanish version by Lobo et al.^44^). This instrument aims to detect individuals with a diagnosable psychiatric disorder^43^. It is composed of 28 items and comprises four subscales: Somatic Symptoms (7 items; i.e. “Been feeling perfectly well and in good health?”), Anxiety/Insomnia (7 items; i.e. “Lost much sleep over worry?”), Social Dysfunction (7 items; i.e. “Been managing to keep yourself busy and occupied?”), and Depression (7 items; i.e. “Been thinking of yourself as a worthless person?”), with 4-point Likert response format (from 0 to 3), progressively worse. Thus, a high score indicates poorer psychological health. Cronbach’s alpha for the four subscales, in this study, were 0.83, 0.89, 0.74, and 0.84 respectively.

*Control variables.* Demographic variables (gender, age, and seniority at the organization) were controlled. Gender was a categorical variable (male = 1, female = 2), age and seniority in the organization were measured as continuous variables. In addition, this study found that sex was associated with mental health and was correlated with PA.

### Statistical analyses

IBM SPSS v. 26 was used to compute descriptive statistics (mean scores, standard deviations, internal consistency, and Pearson correlations). AMOS 24 software was used for analyzing structural equation modeling (SEM) with maximum likelihood estimation of the moderated mediation model, to test the hypothesized theoretical model fit (see Fig. 1).

To test the paths, following recommendations by Collier^45^ for mediated moderation test, we defined path A to test the effect of stress on vigor at work; path B for vigor at work on mental health; path C to test the direct effect; path D for analyzing the interaction between stress × weekly PA on vigor at work; and finally, path F for effects of weekly PA on vigor at work. For computing the path interaction, the independent variable, and the moderator were transformed centering the data, before multiplication. To analyze the indirect effect through vigor at work, as a mediator, we defined the estimate function testing as the product of paths A and B. For testing the weekly PA, as moderator at different levels, we multiplied path D by the standard deviation of the moderator (weekly PA). Next, this value is added to the A path. Finally, the result obtained is multiplied by path B, getting the standard above. To get the standard below we followed the same procedure, but with the negative standard deviation of the moderator (weekly PA)^45^.

The evaluation of the fit of the model was based on the chi-square test (χ^2^) and several indices as Comparative Fit Index (CFI), Normative Fit Index (NFI), Goodness-of-Fit Index (GFI), Adjusted Goodness-of-Fit Index (AGFI), and Rootmean-Square Error of Approximation (RMSEA). The values greater than 0.90 for CFI, NFI, GFI y AGFI indicate an acceptable model fit^46^. For the RMSEA, values less than 0.08 are considered an acceptable model fit^47,48^.

## Results

### Descriptive analysis

Pearson correlations (see Table 1) showed positive and significant relationships between stress and all mental health dimensions (Somatic Symptoms, Anxiety/Insomnia, Social Dysfunction, and Depression), and negative relationships with vigor dimensions, but only significant with the Physical Strength and Cognitive Liveliness. All of the vigor dimensions were negatively and significantly related to all the mental health dimensions. Weekly PA was positive and significantly related to the Physical Strength dimension, and negatively with Somatic Symptoms, Social Dysfunction and Depression dimensions.

**Table 1:** Mean scores, standard deviations and Pearson correlations.

	M	SD	1	2	3	4	5	6	7	8	9	10	11	12
1. Stress	0.94	0.25	–											
2. Weekly PA	2214.92	2005.87	0.022	–										
3. Physical Strength	5.31	1.14	−0.189***	0.162***	–									
4. Cognitive Liveliness	5.62	1.05	−0.228***	0.056	0.589***	–								
5. Emotional Energy	6.00	0.89	−0.082	0.021	0.451***	0.467***	–							
6. Somatic Symptoms	5.74	3.93	0.261***	−0.140**	−0.388***	−0.228***	−0.100*	–						
7. Anxiety/Insomnia	6.10	4.53	0.256***	−0.039	−0.351***	−0.190***	−0.109*	0.664***	–					
8. Social Dysfunction	6.93	2.42	0.154***	−0.111*	−0.410***	−0.297***	−0.243***	0.445***	0.394***	–				
9. Depression	1.27	2.47	0.134**	−0.031	−0.233***	−0.156***	−0.097*	0.305***	0.418***	0.251***	–			
10. Gender	281	53.3	0.060	−0.164***	0.019	−0.041	0.138**	0.249***	0.225***	0.088*	0.059	–		
11. Age	38.23	11.50	0.021	−0.035	0.032	−0.087*	−0.003	−0.024	−0.029	−0.031	−0.094*	0.036	–	
12. Seniority	9.89	9.96	0.007	−0.059	0.014	−0.055	0.015	−0.059	−0.042	−0.043	−0.076	−0.008	0.770***	–

Seniority = number of years as worker in the organization; Gender, male = 1, female = 2, for gender row, in M and SD are referred to the number and percentage of female workers.

*p < 0.05; ** *p* < 0.01; *** *p* < 0.001.

### Structural equation modeling (SEM)

To test the model, we used, as indicator variables, stress and weekly PA; whereas for vigor at work factors were Physical Strength, Cognitive Liveliness, Emotional Energy; and for mental health the factors included were Somatic Symptoms, Anxiety/Insomnia, Social Dysfunction and Depression. The measurement model tested had adequate goodness-of-fit (χ^2^ = 145.82, df = 43, *p* < 0.001; CFI = 0.94, NFI = 0.92, GFI = 0.96, AGFI = 0.91; RMSEA = 0.067, 90% CI of RMSEA = 0.055–0.079). Given that all the indicators obtained fit the data, no modification of the originally proposed model was made. For mediated moderation test the hypothesized structural model goodness-of-fit was also adequate (χ^2^ = 145.90, df = 45, *p* < 0.001; CFI = 0.94, NFI = 0.92, GFI = 0.96, AGFI = 0.92; RMSEA = 0.065, 90% CI of RMSEA = 0.054–0.077).

**Figure 2:**
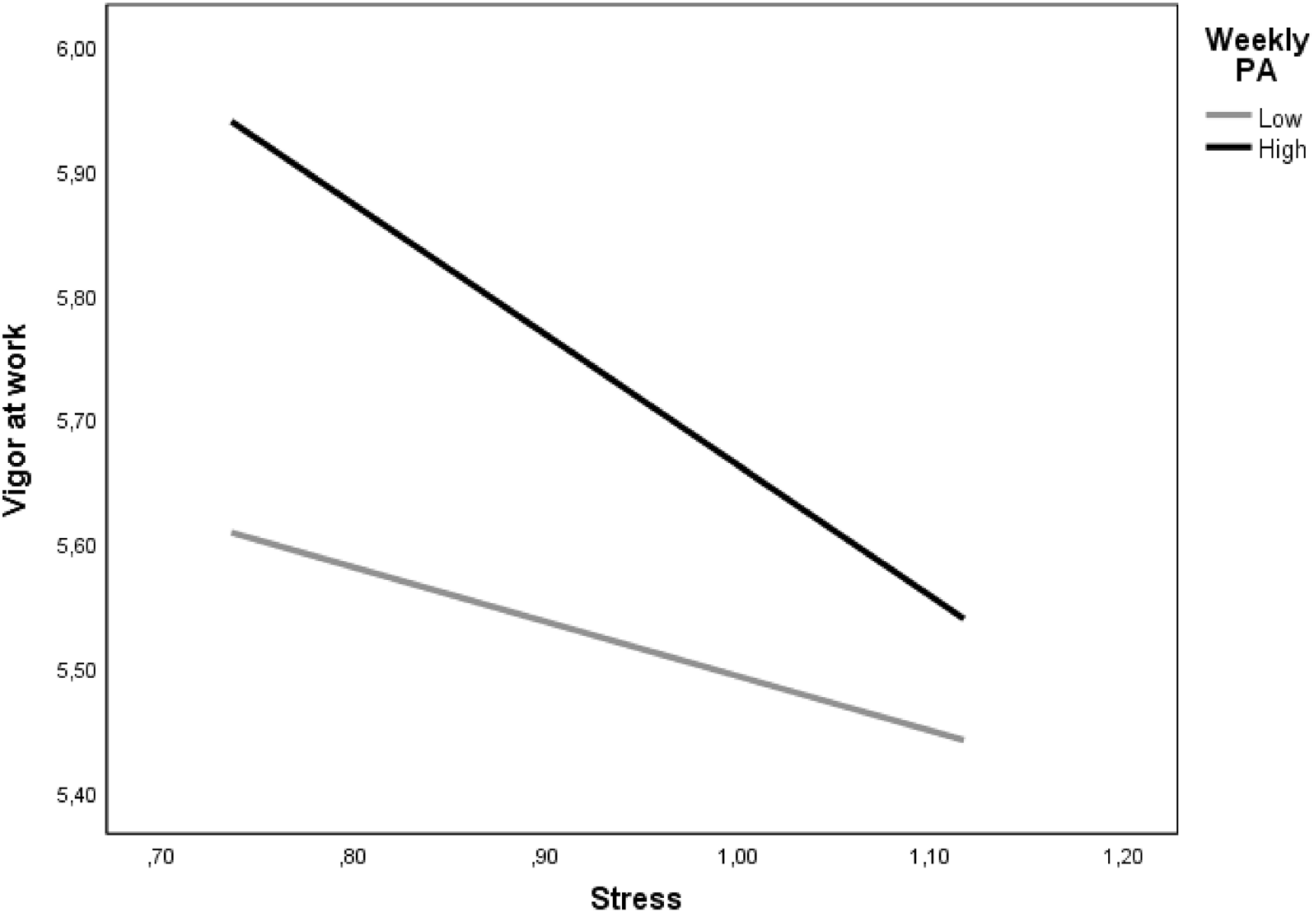
Moderated effect of Weekly PA.

The direct effect of stress on mental health was positive and significant (β = 0.189, *p* < 0.001). Additionally, stress was negatively related to vigor at work (β = -0.234, *p* < 0.001) and vigor at work was negatively related to mental health (β = -0.473, *p* < 0.001). Moreover, weekly PA was positively related to vigor at work (β = 0.182, *p* < 0.001), and the interaction, between stress and PA, was significantly related to vigor at work (β = -0.105, *p* = 0.027). Regarding the mediated indirect effect through vigor at work, moderated by the construct of PA, was (β = 1.57, 95% CI [0.975, 2.373], *p* < 0.001), with a significant moderated mediation index (β = 0.05, 95% CI [0.003, 0.101], *p* = 0.036), using 5,000 bootstrap samples. Thus, weekly PA moderated the relationship between stress and vigor at work. The results showed that when weekly PA is low, the indirect effect is 0.945, still significant (*p* = 0.021). In the same way, when weekly PA is high, the indirect effect is 2.197 and significant (*p* < 0.001) as well (see Table 2). Figure 2 shows a negative difference in the slopes of the regression lines, indicating that weekly PA has a counterproductive effect. Specifically, the negative relationship between stress and vigor at work was greater when doing weekly PA.

**Table 2:** Test for Moderated Mediation Using 95% Confidence Interval.

Direct Relationships		Standardized Coefficient	*t*-values	*p*-**value**
Stress → Vigor at work		−0.234	−4.97	< 0.001
Vigor → Mental health		−0.473	−8.65	< 0.001
Weekly PA → Vigor at work		0.182	3.83	< 0.001
Stress × weekly PA → Vigor at work		−0.105	−2.21	0.027

Moderated indirect relationship	Direct effect	Indirect effect	Confidence interval low/high	*p*-value
Stress → Vigor at work→ Mental health*	0.189 (4.35)	1.571	0.975/2.373	< 0.001
**Probing moderated indirect relationships**				
Low levels of weekly PA		0.945	0.132/1.930	0.021
High levels of weekly PA		2.197	1.393/3.230	< 0.001
**Index of Moderated Mediation**		0.050	0.003/0.101	0.036

* = The indirect effect is moderated by the construct of weekly PA.

Standardized Coefficient reported. Value in parentheses is t-value. Bootstrap Sample = 5,000.

Overall the model accounts for 9.5% of the variance of vigor at work, and 66.2% for Somatic Symptoms, 64.5% for Anxiety/Insomnia, 29.4% for Social Dysfunction, and 19.8% for Depression. The model explained, altogether, 38% of the latent mental health variable.

## Discussion

Due to the importance of stress levels for the mental health in workers, the aim for testing a model was to explore personal resources that can buffer the stress-mental health relationship. For this reason, this study aimed to enhance insight into the relationship between job stress and workers' mental health, testing the fit to the data of a moderated mediation model, where vigor at work mediates the relationship between job stress and workers' mental health, and weekly PA was considered as a potential moderator between stress and vigor at work. In agreement with the hypotheses, the results of the present study confirm the proposed moderated mediation model, where vigor at work mediated the relationship between stress and mental health, while weekly PA moderated the relationship between stress and work vigor.

Regarding the stress-mental health relationship, stress related to lower mental health, since self-reports of mental health indicators suggested an increase in the levels of Somatic Symptoms, Anxiety/Insomnia, Social Dysfunction, and Depression (H1). This result confirms past studies' results. Indeed, a systematic meta-review carried out by Harvey et al.^49^ found that high job demands levels, low control at work and role stress were associated with an increased risk of developing different mental health problems in several studies, with moderate evidence. Thus, Maulik^8^ suggests that workers' mental health should be given more importance, improving the situation at the workplace, promoting a healthy place, and enhancing the wellbeing of all workers. Therefore, it is important to analyze variables that may imply an improvement in the workers´ health. In this vein, we wanted to analyze the role of vigor at work in the stress-mental health relationship. We found that a positive affect, as vigor at work, mediated the relationship between stress and mental health, where stress was negatively related to vigor at work, whereas vigor at work was related to better mental health (H2). These findings supported that stress could lower the energy of workers, reducing their Physical Strength, Cognitive Liveliness, and Emotional Energy from Shirom's perspective of vigor; and not only vigor, concerning energy, resilience, persistence, and effort studied previously by Malik et al.^23^. Also, the vigor-mental health relationship was similar to those found with the levels of depression and insomnia in the studies previously indicated in the introduction. This is a novel result because vigor at work has been mainly related to physical health^50^. Therefore, vigor at work would be one of the explanatory mechanisms by which stress negatively influences mental health. In this venue, promoting a personal resource, such as vigor at work, could help maintaining a better mental health of workers.

Regarding the role of weekly PA in the stress-vigor relationship, employee weekly PA levels moderated the intensity of the indirect relationship between stress-mental health through experienced levels of vigor at work, such that the relationship is more intense and negative when weekly PA levels of employees were higher (H3). Thus, doing high levels of weekly PA increases the negative effect of stress on the levels of vigor. For example, for workers who perceive high levels of stress at work, doing a high weekly PA has a counterproductive role, reducing their levels of vigor at work. Despite Ginoux et al.^51^ found that PA, as a personal resource, improved the levels of vigor; and spending time doing PA had a positive effect on daily recovery^28^ and more energy in workers^52^, the results of the present study showed that this resource may not always have such positive effects. According to Fodor et al.^38^, a different association between stress and PA depending on the type of PA can take place. In particular, they found that vigorous PA (high effort activities, i.e. running) moderated the relationship between demands and exhaustion, but was not significant with moderate PA (it requires effort, but is not exhausting, i.e. easy bicycling). Chekroud et al.^53^, in a sample of more than a million people in the USA, found that doing more PA is not always better, for instance, extreme ranges (more than 23 times per month) or longer time (than 90 min per session) were associated with worse mental health, having a counterproductive role. However, the results of our study go further with the inclusion of stress and other personal energy resource, such as vigor at work. Our results showed that weekly PA boosted the negative effect of stress on vigor at work. It is possible that employees, who perform PA, are consuming part of the resources that would help them to face stress at work by reducing its negative effects on mental health. In this line, if the PA is very intense, exhaustion will probably increase^54^, and vitality and energy could be affected. Hence, it could claim for the health-PA paradox^55^ as it does not always seem to have a protective effect on employees. Holtermann et al.^56^ analyzed the health paradox specifically in work-related and leisure-time PA, founding opposing effects. Particularly, work-related PA increased the risk for long-term sickness absence; whereas leisure time PA decreased this risk. Consequently, the type, duration, and frequency of PA could play an important role in stress-mental health through experienced levels of vigor at work.

Regarding the limitations, the use of a cross-sectional design and a convenience sample prevents the generalization of the results. Therefore, longitudinal studies are needed to further analyze the mechanisms between stress and its relationship with mental health. Additionally, work-related and leisure-time PA should be considered separately, due to its possible paradoxical effect on health^56^. Moreover, it would be interesting, for future studies, to assess the variables of the model at different times, allowing complex moderated mediation models and sequential-type analyses. Also, future research should include other variables that could affect stress levels (i.e. relationships with co-workers and superiors, workplace incivility, verbal abuse at work from co-workers or leaders …) to analyze the stress construct in a more complete way. Another limitation is that we used a self-report measure for the assessment of PA, (IPAQ) which can overestimate the different components of PA^57^, where higher times of moderate-to-vigorous intensity PA have been found on the IPAQ compared to objective measures, as accelerometers^58^. However, Mäder et al.^59^ found that the total PA value reported on the IPAQ correlated with the values offered through accelerometry. Thus, it would be interesting to confirm this model with other PA measures in future research.

The implications of this study are clear. Vigor at work^9,10^ and weekly PA constitute relevant personal resources in the interpretation of the connections between stress and mental health, where changes in stress levels, with the imbalance of psychological demands and control at work^13^, over time, can predict subsequent changes in levels of vigor at work, with weekly PA having an important role. In consequence, vigor at work would decrease the probability of developing mental problems in workers, where weekly PA could have a counterproductive role in employees, consuming part of vigor at work affecting mental health. In addition, the design of preventive and intervention strategies should focus on increasing vigor and control at work, as autonomy and skill development, to reduce stress levels in workers and, this way, improve mental health. Furthermore, it would be necessary to program the type, duration, and frequency of weekly PA to not exceed the threshold, where vigor levels are affected.

## Data Availability

The data that support the findings of this study are available from the corresponding author upon reasonable request.
